# TLR2, TLR4 and TLR10 Shape the Cytokine and Chemokine Release of *H. pylori*-Infected Human DCs

**DOI:** 10.3390/ijms21113897

**Published:** 2020-05-29

**Authors:** Theresa Neuper, Tobias Frauenlob, Muamera Sarajlic, Gernot Posselt, Silja Wessler, Jutta Horejs-Hoeck

**Affiliations:** 1 Department of Biosciences, University of Salzburg, 5020 Salzburg, Austria; theresa.neuper@sbg.ac.at (T.N.); tobias.frauenlob@stud.sbg.ac.at (T.F.); muamera.sarajlic@sbg.ac.at (M.S.); gernot.posselt@sbg.ac.at (G.P.); silja.wessler@sbg.ac.at (S.W.); 2 Cancer Cluster Salzburg (CCS), 5020 Salzburg, Austria

**Keywords:** *Helicobacter pylori*, human conventional dendritic cell (cDC), Toll-like receptor 2/4/10, Type IV secretion system, CagPAI

## Abstract

*Helicobacter pylori (H. pylori)* is a stomach pathogen that persistently colonizes the gastric mucosa, often leading to chronic inflammation and gastric pathologies. Although infection with *H. pylori* is the primary risk factor for gastric cancer, the underlying mechanisms of pathogen persistence and consequential chronic inflammation are still not well understood. Conventional dendritic cells (cDCs), which are among the first immune cells to encounter *H. pylori* in the gastric lining, and the cytokines and chemokines they secrete, contribute to both acute and chronic inflammation. Therefore, this study aimed to unravel the contributions of specific signaling pathways within human CD1c^+^ cDCs (cDC2s) to the composition of secreted cytokines and chemokines in *H. pylori* infection. Here, we show that the type IV secretion system (T4SS) plays only a minor role in *H. pylori*-induced activation of cDC2s. In contrast, Toll-like receptor 4 (TLR4) signaling drives the secretion of inflammatory mediators, including IL-12 and IL-18, while signaling via TLR10 attenuates the release of IL-1β and other inflammatory cytokines upon *H. pylori* infection. The TLR2 pathway significantly blocks the release of CXCL1 and CXCL8, while it promotes the secretion of TNFα and GM-CSF. Taken together, these results highlight how specific TLR-signaling pathways in human cDC2s shape the *H. pylori*-induced cytokine and chemokine milieu, which plays a pivotal role in the onset of an effective immune response.

## 1. Introduction

*Helicobacter pylori (H. pylori)* colonizes the human stomach, and thus the gastric epithelium is the first barrier to encounter the pathogen. Consequently, gastric epithelial cells are primarily infected; however, various innate immune cells comprising macrophages, conventional dendritic cells (cDCs) and neutrophils also reside in the lamina propria of infected individuals. Of note, CD1c^+^ conventional DCs (cDC2s) are known to penetrate the gastric epithelial lining and directly interact with *H. pylori* via their luminal endings [1,2]. Therefore, cDC2s and the ensemble of cytokines and chemokines they secrete are likely to shape the microenvironment of the stomach lining and the subsequent immune response following infection. In this respect, several studies reported that human monocyte-derived DCs (moDCs), murine bone-marrow-derived DCs (BM-DCs), and soluble mediators released by them contribute to the induction of both effector and regulatory T cells in the context of *H. pylori* infection [3,4,5]. Yet, to our knowledge, there are no studies on the response of primary cDC2s to *H. pylori*. Importantly, cDC2s have the advantage that they can be directly isolated from human PBMCs and do not depend on further in vitro differentiation. Furthermore, the fact that cDC2s are among the first immune cells to be infected with *H. pylori*, combined with their unique ability to bridge the innate and adaptive immune responses, emphasizes the need for detailed and comprehensive analysis of *H. pylori*-infected cDC2s.

The type IV secretion system (T4SS) is a sophisticated infection apparatus of *H. pylori* that releases virulence factors and other bacterial products into the host cell (recently reviewed in [6,7,8]). The components of the T4SS are encoded by the so-called cag pathogenicity island (CagPAI), which is a 40-kb sequence comprising 32 genes coding for proteins of the needle-like structure of the T4SS, proteins that interact with surface molecules on the host cell, and virulence factors of *H. pylori*. Once the T4SS is established and engages the host cell, bacterial products such as the oncoprotein CagA and peptidoglycan can be translocated into the host cytoplasm [7]. The importance of the T4SS to *H. pylori* pathogenicity was demonstrated by infection of epithelial cells with an *H. pylori* mutant lacking the CagPAI gene cluster, which resulted in failure to regulate *H. pylori*-specific genes in the epithelial cells [9]. While the T4SS is the main driver of the *H. pylori*-induced phenotype of epithelial cells, information on the role of the T4SS in cDC2s remains scarce, which was an impetus for this study.

A second focus of the study is the impact of TLR signaling in *H. pylori*-infected cDC2s. cDC2s are immune sentinels, sampling various human tissues for foreign substances [10]; therefore, they are well equipped with various TLRs. The TLR family comprises 10 members, TLR1–10, all of which are expressed by cDC2s, with the exception of TLR9, which is predominately expressed on plasmacytoid DCs (reviewed in [11]). TLRs enable cDC2s to recognize a wide variety of microorganisms and alert the host, via the adaptor molecules MyD88 or TRIF, to the presence of potentially harmful invaders [12]. Although *H. pylori* has been reported to be very effective in evading TLR recognition [13,14], results of early studies using MyD88-deficient mice suggested a crucial role for TLR signaling during *H. pylori* infection, as activation of MyD88-deficient BM-DCs upon *H. pylori* infection is profoundly diminished compared to wild-type cells [15].

This study aimed to investigate the importance of two characteristic processes during infection of cDC2s by *H. pylori*: (1) the influence of the T4SS and (2) the role of TLR signaling in the composition of the cytokines and chemokines released by cDC2s in response to *H. pylori*. Analysis of an *H. pylori* mutant lacking the T4SS and antibody-based inhibition of TLR2, TLR4 or TLR10, respectively, revealed that the impact of the T4SS on cDC2 activation is minor compared to the contribution of TLR signaling. TLR4 signaling drives the *H. pylori*-induced secretion of inflammatory cytokines, whereas TLR10 seems to act in an immuno-modulatory manner upon *H. pylori* infection of cDC2s. Interestingly, the effects of TLR2 appear to be Janus-faced, as TLR2 signaling inhibits chemoattractants on the one hand but promotes inflammatory cytokines on the other.

## 2. Results

### 2.1. The Type IV Secretion System Plays Only a Minor Role during Infection of Human CD1c^+^ Conventional DCs (cDC2s) by H. pylori

**Figure 1:**
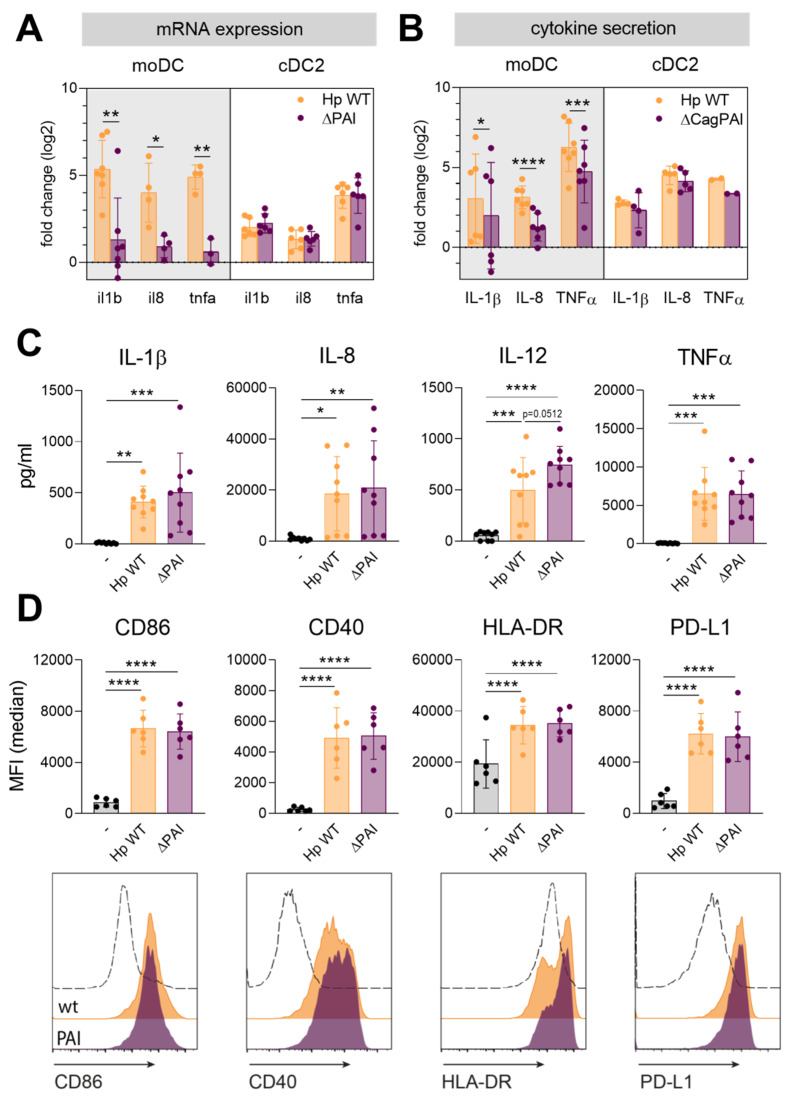
Activation of CD1c^+^ conventional DC (cDC2s) is similar upon infection with *Helicobacter pylori* wt or a mutant lacking the T4SS. (**A**,**B**) Monocyte-derived DCs (moDCs) or cDC2s were infected with *H. pylori* wt or a mutant lacking the type IV secretion system (ΔPAI) at a multiplicity of infection (MOI) of 5. One hour post-infection, mRNA expression was analyzed by qPCR (**A**). After 4 h, cytokine secretion was evaluated by ELISA or multiplex technology (**B**). Log2 fold changes compared to the untreated sample are shown. For comparing fold changes of Hp wt and PAI-infected samples, a paired t-test was performed. (**C**) Cytokine and chemokine secretion by cDC2s was measured by multiplex technology 16 h post-infection. (**D**) Surface marker expression was monitored by flow cytometry. Median fluorescence intensity of six donors (upper panel) and histograms of one representative donor (lower panel) are shown. Dots represent individual donors, bars show means ± SDs. For statistical analysis, repeated-measures, one-way ANOVA with Tukey’s post-hoc test was performed. (* *p* ≤ 0.05, ** *p* ≤ 0.01, *** *p* ≤ 0.001, **** *p* ≤ 0.0001).

In order to identify the contribution of the Type IV secretion system (T4SS) to *H. pylori*-induced activation of DCs, we monitored DC maturation upon stimulation with a mutant lacking the pathogenicity island gene cluster (ΔPAI) compared to the wild-type (*H. pylori* wt) strain. Because in the past decades *in vitro*-differentiated moDCs have been commonly used to investigate human DC biology in the course of *H. pylori* infection, we first compared the immune responses of moDCs and cDC2s, which were directly isolated from the blood. We assessed DC activation by monitoring cytokine and chemokine mRNA expression and protein secretion and by investigating the expression levels of co-stimulatory and co-inhibitory surface molecules (Figure 1 and Figure S1).

Analysis of cytokine expression and secretion at early time points revealed that the T4SS significantly contributes to the expression and release of the pro-inflammatory cytokines IL-1β, CXCL8/IL-8, and TNFα in moDCs (Figure 1A,B), while the secretion upon stimulation with both strains converges at later time points (Figure S1A). In contrast, cDC2s express and release similar levels of these mediators upon infection with the *H. pylori* wt and the ΔPAI mutant strain at early and later time points (Figure 1A–C).

We also analyzed the expression of co-stimulatory proteins (CD40, CD86), antigen-presenting machinery (HLA-DR), and co-inhibitory molecules (PD-L1). The expression of all tested surface molecules increased to the same extent upon infection with either wt *H. pylori* or the ΔPAI mutant in cDC2s and moDCs (Figure 1D and Figure S1B). This set of data indicates that the T4SS might contribute to the release of inflammatory cytokines immediately post-infection in moDCs, whereas it does not play a crucial role in the secretion of IL-1β, IL-8, and TNFα and in surface marker expression at later time points. Additionally, the T4SS in general plays only a minor role in the course of *H. pylori* infection of cDC2s. The observed differences between moDCs and cDC2s, which are probably the more common and relevant DC subtype in the gastric lining, emphasize the need for comprehensive analysis of signals contributing to the activation of cDC2s upon *H. pylori* infection.

### 2.2. Contribution of Toll-Like Receptors (TLRs) to H. pylori-Induced Cytokine and Chemokine Secretion by Human cDC2s

**Figure 2:**
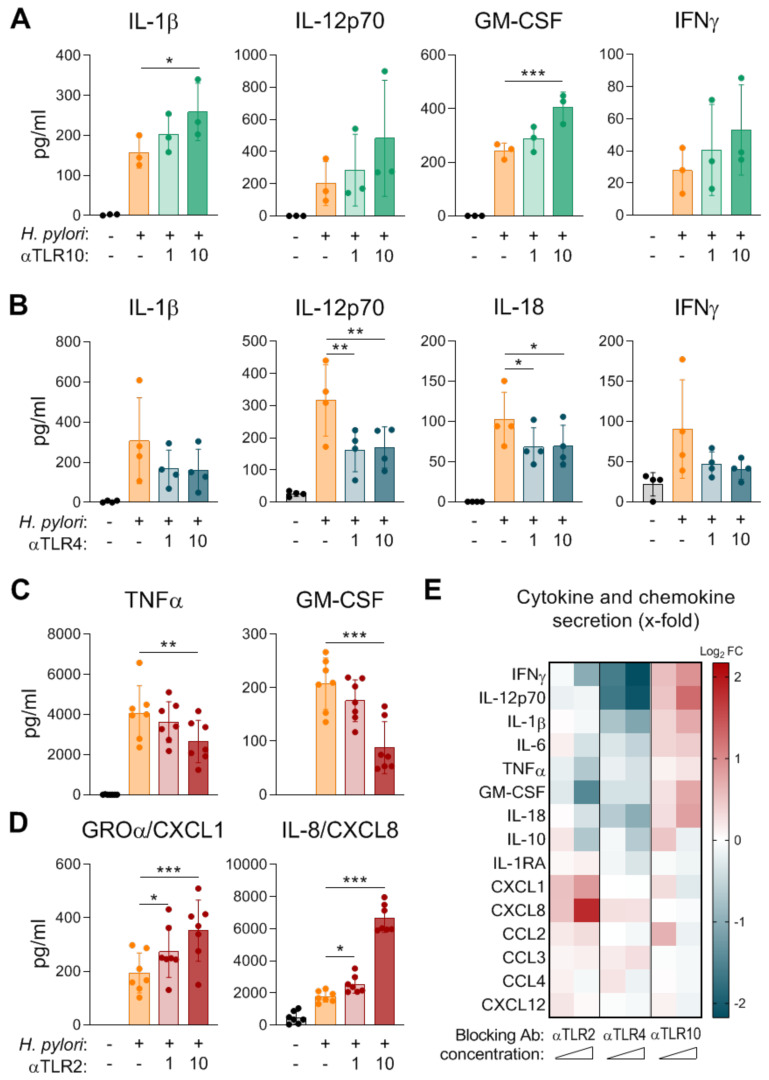
Impacts of TLR2, TLR4, and TLR10 on cytokine and chemokine secretion by cDC2s in response to *H. pylori*. cDC2s were infected with *H. pylori* P12 (MOI 5). Twenty minutes prior to infection, the cDC2s were treated with blocking antibodies to (**A**) TLR10, (**B**) TLR4, or (**C**,**D**) TLR2 at a concentration of 1 µg/mL or 10 µg/mL. Cytokine and chemokine secretion was monitored 16 h post *H. pylori* infection by multiplex assay. Dots represent individual donors, bars and lines show means ± SDs. For statistical analysis, repeated-measures ANOVA with Dunnett’s post-hoc test was performed. (* *p* ≤ 0.05, ** *p* ≤ 0.01, *** *p* ≤ 0.001). (**E**) Log2 fold change (Log_2_ FC) was calculated using log2 of the following ratio, mean of inhibitor-treated samples:mean of *H. pylori*-infected samples. Red: up-regulation, blue: down-regulation.

Given the minor impact of T4SS signaling on cDC2 activation that we observed and the fact that TLR signaling is known to be activated during *H. pylori* infection, we investigated the contribution of TLRs to *H. pylori*-induced secretion of cytokines and chemokines in cDC2s. Several publications along these lines described involvement of TLR2, TLR4, and TLR10 signaling in the context of *H. pylori* infection in epithelial cells [16,17,18]. However, studies investigating TLRs, especially TLR10, in immune cells, are rare. To unravel the impacts of TLR2, TLR4, and TLR10 signaling during *H. pylori* infection of human cDC2s, we treated cDC2s with blocking antibodies targeting TLR2, TLR4, or TLR10 prior to *H. pylori* infection. Inhibition of TLR10 resulted in enhanced *H. pylori*-induced release of pro-inflammatory cytokines, including IL-1β, IL-12, GM-CSF, and IFNγ (Figure 2A,E), while treatment with blocking antibodies in the absence of *H. pylori* did not alter the DC phenotype (Figure S2). In contrast to TLR10 blockade, inhibition of TLR4 specifically dampened the secretion of Th1/Th17-related pro-inflammatory cytokines, including IL-1β, IL-12, IL-18, and IFNγ (Figure 2B,E). Other cytokines and chemokines were not significantly affected by inhibition of TLR4 or TLR10 (Figures S3 and S4). Blocking TLR2 signaling seems to have dual effects in the context of *H. pylori*-infected cDC2s (Figure 2C,D). While TNFα and GM-CSF levels were significantly decreased in the presence of a TLR2 blocking antibody (Figure 2C), the secretion of CXCL1 and CXCL8 in response to *H. pylori* was significantly enhanced (Figure 2D). All other measured cytokines and chemokines were not considerably altered by inhibition of TLR2 signaling (Figure S5). As visualized in Figure 2E, our data indicate that TLR10 acts in an immuno-modulatory manner, because TLR10 inhibition results in increased induction of numerous cytokines in cDC2s. In contrast, we observed diminished levels of primarily inflammatory cytokines upon TLR4 blocking; thus, we propose that TLR4 is a driver of the inflammatory response during *H. pylori* infection of cDC2s (Figure 2E). Interestingly, TLR2 drives inflammation by promoting the release of inflammatory cytokines on the one hand and seems to dampen chemotaxis by blocking CXCL1 and CXCL8 secretion on the other hand (Figure 2E).

## 3. Discussion

*H. pylori* is the leading cause of gastric cancer and the only bacterial type I carcinogen listed by the WHO in the context of stomach cancer. One hallmark of *H. pylori* infection is chronic inflammation as a result of an immune response that fails to clear the pathogen. This emphasizes the importance of unraveling how *H. pylori* activates specific branches of the immune system. DCs play a central role in orchestrating immune responses by bridging the innate and adaptive immune systems. Therefore, this study aimed to explicate pattern recognition receptors contributing to the *H. pylori*-induced DC phenotype. To date, all studies investigating *H. pylori* infection of human DCs have been performed in human moDCs. However, CD1c^+^ DCs, which are predominantly found in gastric tissue, are clearly distinct from moDCs, as they arise from a different precursor [19]. Thus, we believe that CD1c^+^ cDC2s, which are isolated directly from blood and do not depend on further in vitro differentiation from monocytes, are an important model to improve our understanding of *H. pylori* infection of human DCs.

While several studies investigated the contributions of two *H. pylori* products, namely, vacuolating toxin A (VacA) and γ-glutamyltranspeptidase, to the induction of a tolerogenic DC phenotype in moDCs and BM-DCs [20,21,22], information on the role of CagPAI during DC infection is scarce. We found that cDC2s, and at later time points also moDCs, secrete similar levels of IL-1β, IL-8, and TNFα upon infection with the wt and the CagPAI-deficient strain. This is in line with a study where infection of DCs with CagPAI positive and negative *H. pylori* strains resulted in the production of similar levels of most pro-inflammatory cytokines [23]. In contrast, infection of murine DCs with a mutant lacking CagPAI resulted in reduced levels of IL-1β after overnight treatment. We did not observe decreased IL-1β 16 h post-infection, but this difference might be explained by the different durations of infection, or it could be a consequence of species-specific differences in DC function, which furthers emphasizes the advantage of using human cDC2s [24]. However, it has been reported that infection of epithelial cells with a CagPAI-deficient *H. pylori* mutant reflects the phenotype of a mock-treated cell rather than the phenotype of an *H. pylori*-infected cell [9], which does not apply to cDC2s. This might be a result of differences in TLR expression levels in epithelial cells and cDC2s. Accordingly, it has been shown that while TLR negative HEK293 cells do not secrete pro-inflammatory cytokines upon infection with a ΔCagPAI mutant, overexpression of TLRs prior to infection enables cytokine secretion [25].

While TLRs have been investigated intensively for decades, TLR10 remains the least-studied member, as its ligand is still unknown. In a model of septic shock, TLR10 transgenic mice exhibited a suppressed inflammatory response upon LPS administration, indicating immuno-modulatory functions for this receptor [26]. Additionally, recent literature suggests that *H. pylori* induces the expression of TLR10 and also signals via TLR10 in epithelial cells [17,27]. Moreover, polymorphisms in the TLR10 gene might influence susceptibility to *H. pylori* [28,29]. Our studies revealed that blocking TLR10 results in enhanced levels of IL-1β and GM-CSF, indicating that TLR10 might ameliorate immune responses (Figure 2A). The anti-inflammatory capacities of TLR10 have already been highlighted in a model using pam3CSK4 (a synthetic tri-acylated lipopeptide) to induce inflammation, wherein the authors observed increased levels of IL-1β, IL-6, IL-8, and TNFα upon inhibition of TLR10 in human PBMCs [30].

Much effort has been made to characterize how *H. pylori* modifies its LPS to evade recognition by TLR4 [13,14]. However, human cDC2s seem to be able to recognize *H. pylori* via TLR4, as we can show that administration of a blocking antibody to TLR4 prior to *H. pylori* infection results in diminished secretion of inflammatory cytokines in general and significantly reduced levels of IL-12 and IL-18 in particular (Figure 2B). In accordance with our observations, Kaebisch et al. reported that blocking TLR4 in human moDCs dampens the *H. pylori*-induced secretion of IL-6, IL-10, and IL-12 [31]. Based on our data, we propose that TLR4 signaling in cDC2s might be important for bacterial clearance by driving inflammatory T cell responses toward *H. pylori*. Accordingly, a study revealed that blocking TLR4 in a mouse model led to a higher bacterial load and higher gastritis score but significantly lower levels of the Th1-associated cytokine IL-12 upon *H. pylori* infection [32].

A contribution of TLR2 during *H. pylori* infection has been suggested by a study comparing *Acinetobacter lwoffii* and *H. pylori* infection. The authors observed enhanced TLR2 expression and more regulatory T cells in response to *H. pylori* compared to *A. lwoffii* and thus proposed that TLR2 might be involved in induction of tolerance [33]. The role of TLR2 during *H. pylori* infection has been further analyzed using TLR2 knockout (TLR2ko) mice [34,35,36]. These studies showed that TLR2ko mice display a reduced *H. pylori* load, increased neutrophil infiltration, and higher levels of IFNγ-expressing T cells compared to TLR2 wt mice. The increase in neutrophil infiltration reported by Sun et al., might be explained by our finding that TLR2 inhibition significantly amplified the release of the chemoattractants CXCL1 and CXCL8 (Figure 2D). Additionally, the authors also showed significant decreases in IL-6, IL-12, IL-23, and TNFα release in *H. pylori*-infected murine BM-DCs from TLR2ko mice [36]. We observed the same effect, at least for TNFα, when we blocked TLR2 in human cDC2s during *H. pylori* infection, where TNFα levels were significantly diminished (Figure 2C).

Taken together, our study highlights that primary cDC2s are a useful model to study the biology and activation of human DCs upon infection with *H. pylori*. We demonstrate that TLR4 signaling promotes the release of inflammatory cytokines by human cDC2s upon *H. pylori* infection. In contrast, TLR10 may act in an immuno-modulatory manner by attenuating the secretion of inflammatory mediators. TLR2 plays a dual role, as TLR2 promotes the release of GM-CSF and TNFα but dampens CXCL1 and CXCL8 levels upon *H. pylori* infection. These data provide insights into how specific TLR pathways shape the *H. pylori*-induced cytokine and chemokine milieu produced by human cDC2s. Since TLR-mediated activation of DCs plays a pivotal role in the initiation of effective, adaptive immune responses, our findings might contribute to a better understanding of the complex host defense against *H. pylori*.

## 4. Materials and Methods

### 4.1. Isolation of Primary CD1c^+^ Dendritic Cells (cDC2s)

This study was performed in agreement with the guidelines of the World Medical Association’s Declaration of Helsinki. No additional approval by the local ethics committee was required given that national regulations do not require informed consent in the case of anonymous blood cells being discarded after plasmapheresis (buffy coats). Buffy coats of healthy individuals were obtained from the Blood Bank Salzburg, Austria. After density-gradient isolation of peripheral blood mononuclear cells (PBMCs) using Histopaque-1077 (Sigma-Aldrich, Vienna, Austria), erythrocytes were lysed using ACK buffer (150 mM NH_4_Cl, 10 mM KHCO_3_, 0.1 mM EDTA, pH 7.4). Following three washing steps, PBMCs were used for magnetic cell separation (CD1c (BCDA-1)^+^ Dendritic Cell Isolation Kit, Miltenyi Biotec, Bergisch Gladbach, Germany) to isolate cDC2s. According to manufacturer’s protocol, 1 × 10^8^ PBMCs were resuspended in 100 µL MACS buffer (PBS with 0.5% BSA, 2mM EDTA) and 100 µL FcR blocking reagent, 100 µL CD19 microbeads, and 100 µL of CD1c-biotin were added and incubated for 15 min at 4 °C. To provide positive separation of primary dendritic cells, CD1c^+^ B-cells were depleted by using an LD column and collecting just the unlabeled fraction. Washing was performed by adding buffer and centrifugation at 300× *g*, 4 °C, 10 min. Subsequently, cells were resuspended in 400 µL MACS buffer and 100 µL anti-biotin microbeads were added to facilitate magnetic separation of cDC2s using MS columns. Three washing steps with 500 µL MACS buffer were performed, before cDC2s were seeded in RPMI 1640 medium containing 10% heat-inactivated FCS, 1% L-glutamine and used for further experiments.

### 4.2. Generation of moDCs

PBMCs were seeded in moDC medium (RPMI 1640 medium containing 10% heat-inactivated FCS, 1% L-glutamine, 50 mM β-mercaptoethanol) at a density of 10^7^/mL for 70 min. After monocyte adherence, cells were washed four times before moDC medium supplemented with IL-4 and GM-CSF (50 ng/mL each) was added to the monocytes. On day 3 of differentiation, fresh medium containing IL-4 and GM-CSF was added. On day 7 of differentiation, moDCs were used for further experiments.

### 4.3. Bacterial Culture and Infection Experiments

All *H. pylori* strains (*H. pylori* wild-type P12 (wt) and P12 ΔcagPAI) were cultured under microaerophilic conditions (CampyGen Atmosphere Generation Systems, Thermo Scientific, Vienna Austria) and at 37 °C on GC agar plates (10% horse serum). The mutant strain P12 ΔcagPAI is kanamycin resistant, and thus selective GC agar plates supplemented with kanamycin (8 µg/mL) were used to cultivate P12 ΔcagPAI. For infection of cDC2s, *H. pylori* was harvested in PBS and added to the cells at a multiplicity of infection (MOI) of 5 for the indicated time points. For TLR inhibition experiments, primary DCs were treated with α-TLR2 (PAb-hTLR2, InvivoGen, Toulouse, France), α-TLR4 (W7C11, α-hTLR4-IgG, InvivoGen, Toulouse, France), or α-TLR10 (3C10C5, Abcam, Cambridge, UK) at a concentration of 1 µg/mL or 10 µg/mL 20 min prior to infection.

### 4.4. Flow Cytometry

For analysis of surface marker expression by flow cytometry, cells were harvested in FACS buffer (1% BSA, 2mM EDTA) and transferred to a 96-well v-bottom plate. For surface marker staining, all antibodies were mixed in PBS and 30 µL staining mix was added per well. Staining was performed for 30 min at 4 °C in the dark. Thereafter, cells were washed once and fixed in 4% PFA for 15 min at room temperature following two washing steps with FACS buffer. A FACS Canto II flow cytometer (BD Biosciences, San Francisco, CA, USA) was used to assess median fluorescence intensities (MFI) of single, living CD1c^+^ primary DCs using the following antibodies: CD1c-BV421 (L161, Biolegend, Biozym, Vienna, Austria), Fixable Viability Dye-eFluor506 (eBioscience, Thermo Fisher Scientific, Vienna, Austria), CD40-FITC (5C3, eBioscience), CD86-PE (IT2.2, eBioscience), HLA-DR-APC (LN3, eBioscience), CD14-PerCP-Cy5.5 (MɸP9, BD), PD-L1-PE-Cy7 (MIH1, BD). Data analysis was performed on FlowJo 10 Software.

### 4.5. Multiplex Assay

Cytokine and chemokine secretion was monitored by multiplex assay using the Cytokine/Chemokine/Growth Factor 45-Plex Human ProcartaPlex™ from ThermoFisher Scientific, Vienna, Austria. For multiplex bead preparation, beads were washed once in washing buffer before they were re-suspended in assay buffer and distributed into a 96-well V-bottom plate (8.34 µL per well). Thereafter, standards and samples (15 µL) were added to the respective wells. All of the following incubation steps were conducted on an orbital shaker (300 rpm). After incubation (4 °C, overnight), the plate was washed three times and incubated with detection antibody solution for 30 min at room temperature (15 µL per well). Subsequently, three washing steps were performed before 20 µL of streptavidin-PE solution (1:1 in assay buffer) was added to each well and incubated for 30 min at room temperature. Again, three washing steps were performed. For analysis, samples were re-suspended in drive fluid and measured on a Luminex Magpix instrument and data were analyzed using Procarta Plex Analyst Software (Thermo Fisher Scientific).

### 4.6. ELISA

Cell supernatants were collected and used for the detection of cytokines by means of sandwich ELISA. Using ELISA kits for IL-1β, IL-8 and TNFα (Peprotech, Eubio, Vienna, Austria) cytokine secretion was measured according to the manufacturer’s instructions. Briefly, the capture antibody was diluted to the given concentration and Nunc MaxiSorp plates (eBioscience, Vienna, Austria) were coated over night at 4 °C. Blocking was performed with PBS containing 1% BSA for 1 h at room temperature. Thereafter, standards and samples were incubated for 2 h at room temperature. After washing, biotinylated detection antibodies were added for 2 h. HRP-labeled streptavidin (R&D, Eubio, Vienna, Austria) was added to the plates for 30 min and the development was performed using TMB substrate (3,3′,5,5′-teramethylbenzidine, Sigma-Aldrich, Vienna, Austria). The TMB reaction was stopped with 2 M H2SO4 and absorbance was measured at 450 nm with a Tecan Infinite 2000 (Tecan, Grödig, Austria). To subtract high background signals, a reference measurement at 650 nm was performed.

### 4.7. Statistics

In the figures, dots represent independent donors and bars indicate means ± standard deviations (SD). Statistical analyses were performed with GraphPad Prism 8 software. Differences between two groups from the same donors were analyzed using paired *t*-test. Multiple groups from the same donors were analyzed using repeated-measures, one-way ANOVA including appropriate post-hoc tests indicated in the figure legends. *p* values <0.05 were considered significant (* *p* ≤ 0.05, ** *p* ≤ 0.01, *** *p* ≤ 0.001, **** *p* ≤ 0.0001).

## 5. Conclusions

In conclusion, this study provides new insights into the role of TLRs and their function as mediators of the *H. pylori*-induced cytokine and chemokine milieu and highlights that primary cDC2s are a useful model to study the activation of human DCs upon *H. pylori* infection.

## Supplementary Materials

Supplementary materials can be found at https://www.mdpi.com/1422-0067/21/11/3897/s1. Figure S1: No changes in the phenotype of moDCs infected with *H. pylori* wt or a mutant lacking the T4SS after 24 h. Figure S2: In the absence of *H. pylori* infection, TLR blocking does not affect the cDC2 phenotype. Figure S3: Effects of TLR10 blocking on *H. pylori*-induced cytokine and chemokine secretion by cDC2s. Figure S4: Effects of TLR4 blocking on *H. pylori*-induced cytokine and chemokine secretion by cDC2s. Figure S5: Effects of TLR2 blocking on *H. pylori*-induced cytokine and chemokine secretion by cDC2s.

## Abbreviations

CagPAI	Cag pathogenicity island
CCL	CC chemokine ligand
CD	Cluster of differentiation
cDC	Conventional dendritic cell
CXCL	CXC-motif ligand
GM-CSF	Granulocyte macrophage stimulating factor
*H. pylori*	*Helicobacter pylori*
IL	Interleukin
LPS	Lipopolysaccharide
PD-L1	Programmed-death ligand 1
TNF	Tumor necrosis factor
TLR	Toll.like receptor
T4SS	Type IV secretion system
