# Supplementary material for: TLR2, TLR4 and TLR10 Shape the Cytokine and Chemokine Release of H. pylori-Infected Human DCs
Source: Int J Mol Sci. 2020 May 29;21(11):3897. doi: 10.3390/ijms21113897 (PMC7311968; doi:10.3390/ijms21113897)
Supplement: Supplementary file 1 [file ijms-21-03897-s001.pdf]

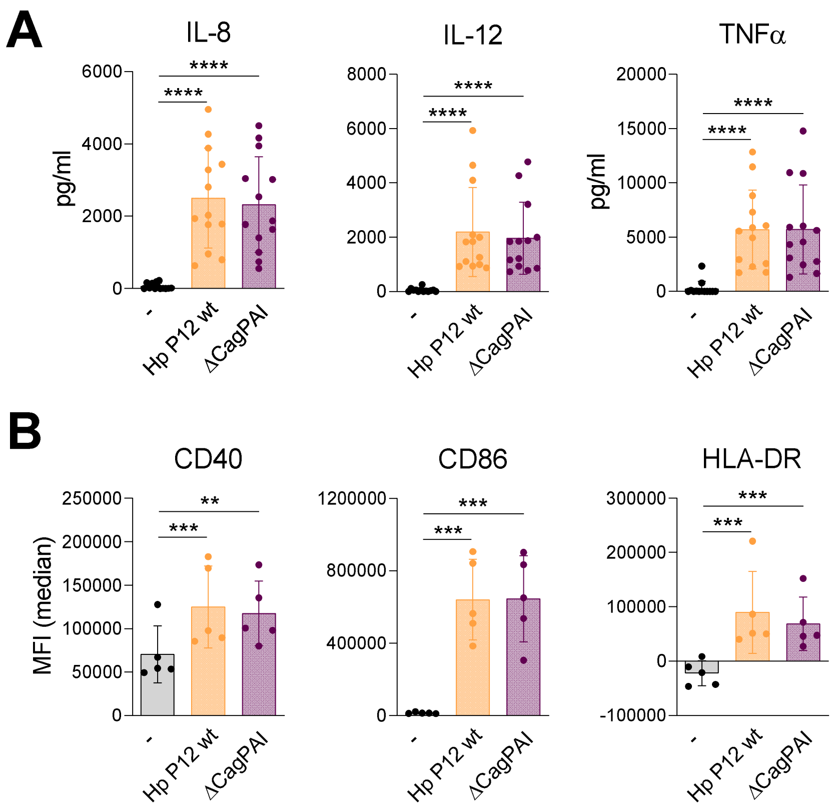

Figure S1 No changes in the phenotype of moDCs infected with *H. pylori* wt or a mutant lacking the T4SS after 24 hours. A/B moDCs were infected for 24 hours with *H. pylori* wt or a mutant lacking the type IV secretion system (ΔCagPAI) at an MOI of 5. Cytokine secretion and surface marker expression were monitored by ELISA and flow cytometry, respectively. Dots represent individual donors, bars show mean±SD. For statistical analysis, repeated measures, one-way ANOVA with Tukey's post-hoc test was performed.

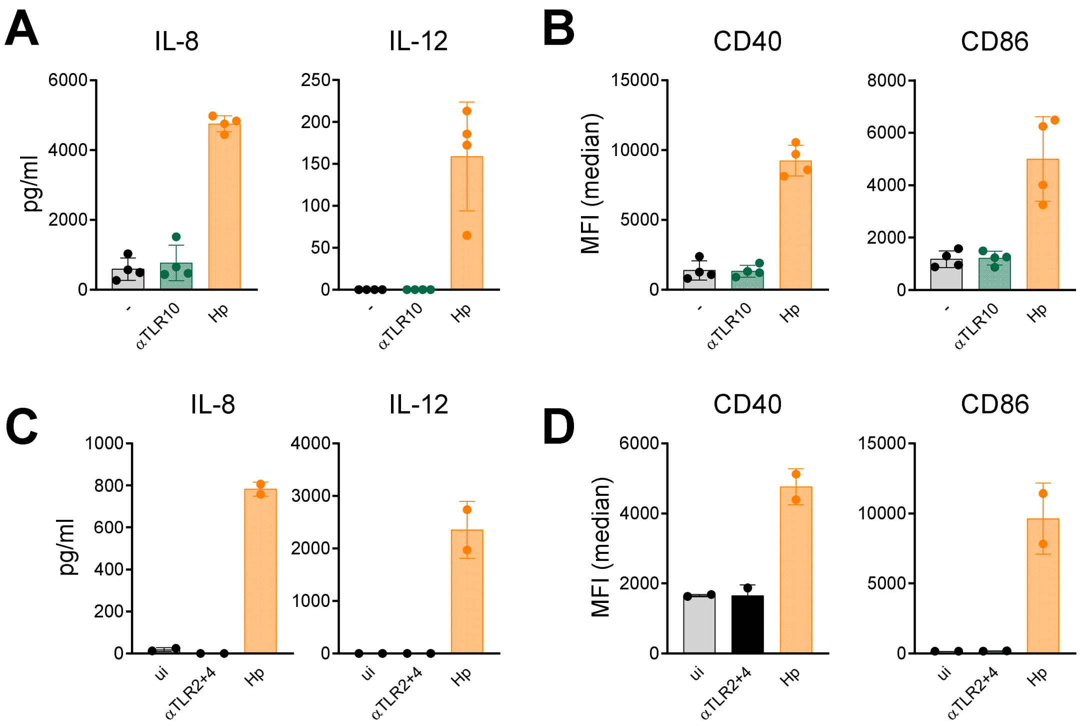

Figure S2 In the absence of *H. pylori* infection, TLR blocking does not affect the cDC2 phenotype. A/B cDC2s were treated with blocking antibody (10 µg/mL) against TLR10 for 16 hours. Infection with *H. pylori* served as a positive control for DC activation. IL-8 and IL-12 secretion was measured by Multiplex-Technology (A) and CD40 and CD86 expression by flow cytometry (B). **C/D** moDCs were treated with blocking antibody (10 µg/mL) against TLR2 and TLR4. Infection with *H. pylori* served as a positive control for DC activation. IL-8 and IL-12 secretion was measured by ELISA (C) and CD40 and CD86 expression by flow cytometry (D). Dots represent individual donors, bars show mean±SD.

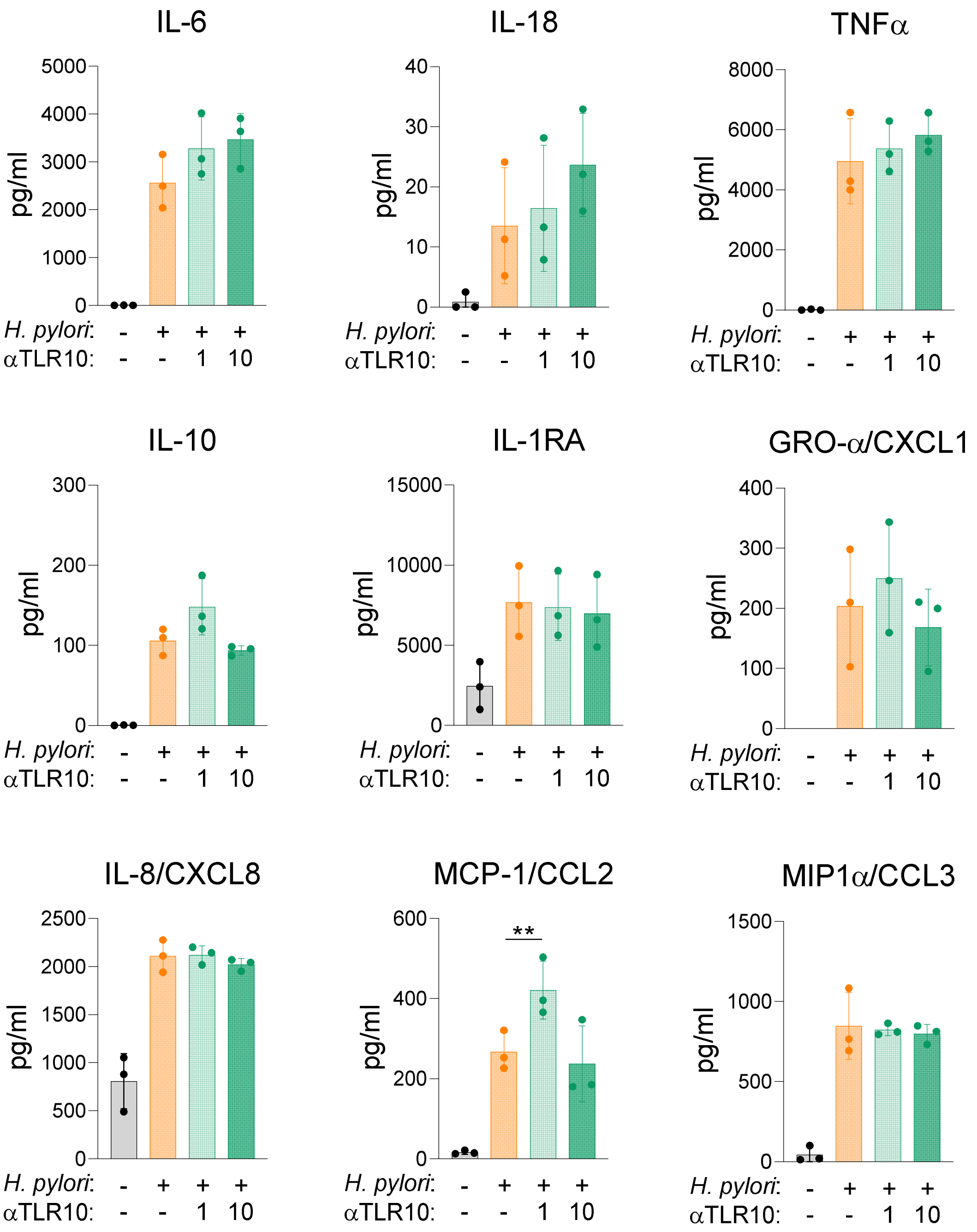

Figure S3 Effects of TLR10 blocking on *H. pylori*–induced cytokine and chemokine secretion by cDC2s. cDC2s were infected with *H. pylori* P12 (MOI 5). 20 minutes prior to infection, cells were treated with a TLR10 blocking antibody at a concentration of 1 µg/ml or 10 µg/ml. Cytokine and chemokine secretion was monitored 16 hours post *H. pylori* infection by Multiplex-Assay. Dots represent individual donors, bars and lines show mean±SD. For statistical analysis ANOVA with Dunnett's post-hoc test was performed. (** $p \leq 0.01$)

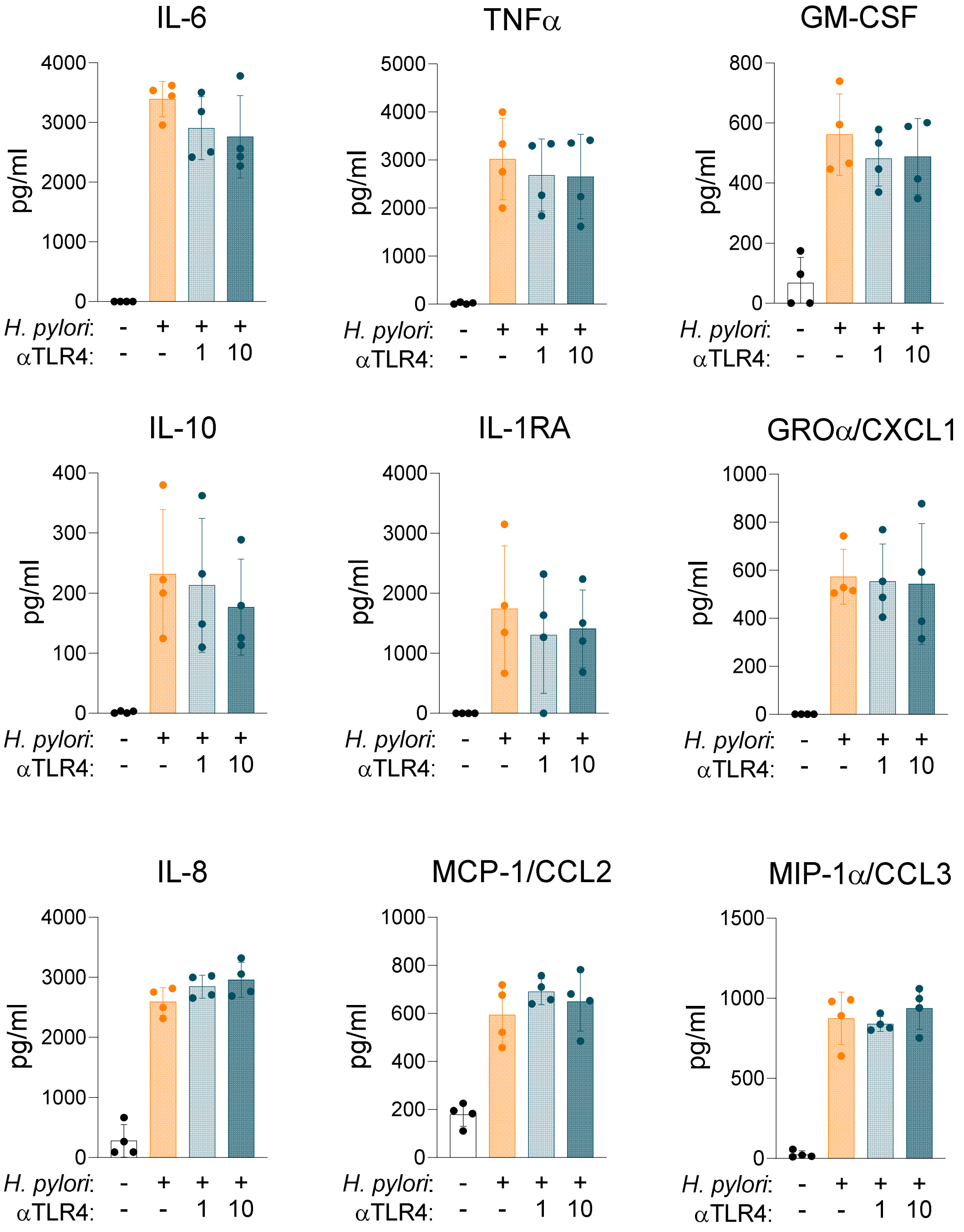

Figure S4 Effects of TLR4 blocking on *H. pylori*-induced cytokine and chemokine secretion by cDC2s. cDC2s were infected with *H. pylori* P12 (MOI 5). 20 minutes prior to infection, cells were treated with a TLR4 blocking antibody at a concentration of 1 µg/ml or 10 µg/ml. Cytokine and chemokine secretion was monitored 16 hours post *H. pylori* infection by Multiplex-Assay. Dots represent individual donors, bars and lines show mean±SD.

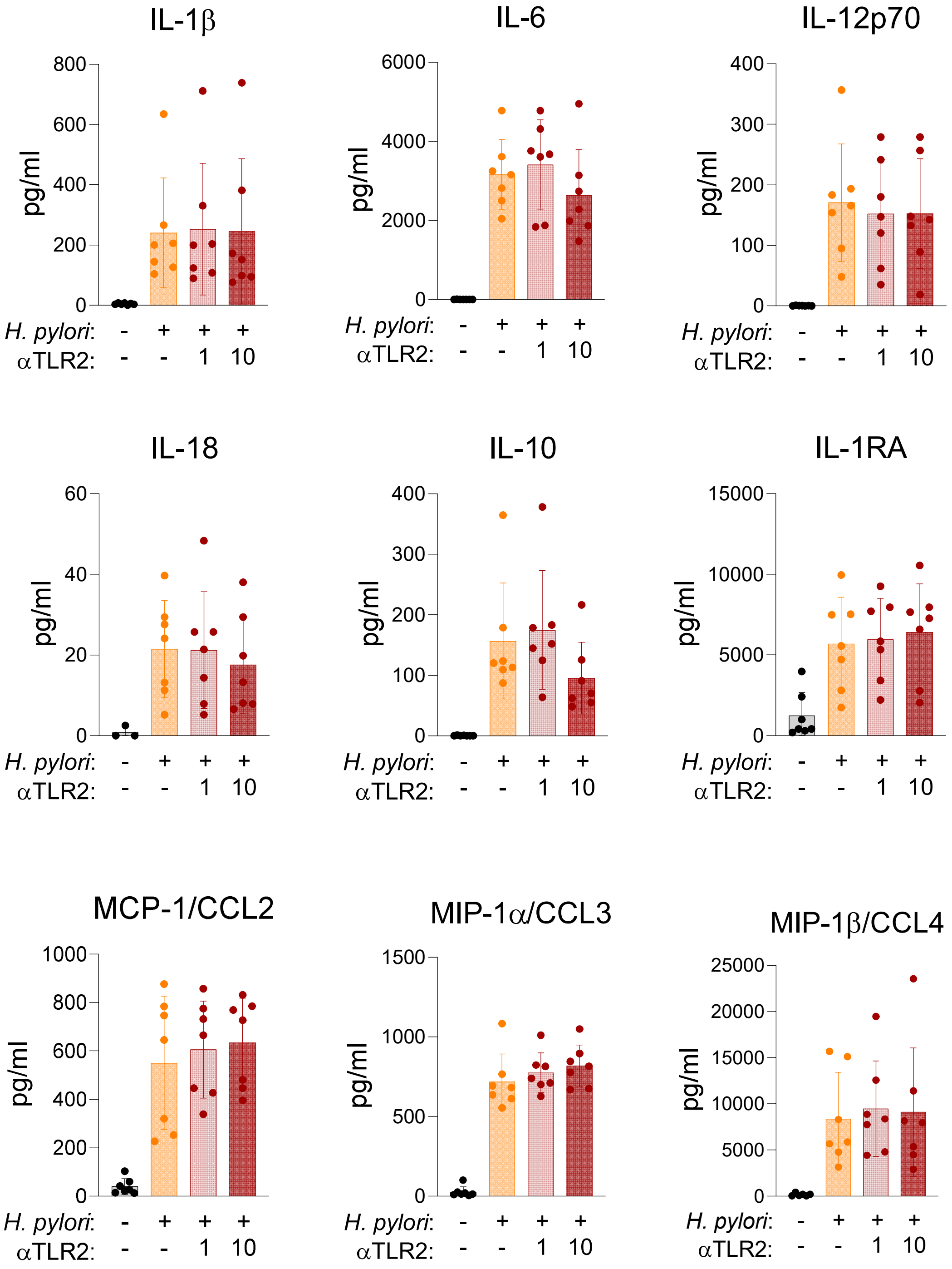

Figure S5 Effects of TLR2 blocking on *H. pylori*-induced cytokine and chemokine secretion by cDC2s. cDC2s were infected with *H. pylori* P12 (MOI 5). 20 minutes prior to infection, cells were treated with a TLR2 blocking antibody at a concentration of 1 µg/ml or 10 µg/ml. Cytokine and chemokine secretion was monitored 16 hours post *H. pylori* infection by Multiplex-Assay. Dots represent individual donors, bars and lines show mean±SD.
